# Ganoderic Acid A Metabolites and Their Metabolic Kinetics

**DOI:** 10.3389/fphar.2017.00101

**Published:** 2017-03-07

**Authors:** Fang-Rui Cao, Li Feng, Lin-Hu Ye, Li-Sha Wang, Bing-Xin Xiao, Xue Tao, Qi Chang

**Affiliations:** Key Laboratory of Bioactive Substances and Resources Utilization of Chinese Herbal Medicine, Ministry of Education, Institute of Medicinal Plant Development, Chinese Academy of Medical Sciences and Peking Union Medical CollegeBeijing, China

**Keywords:** ganoderic acid A, metabolites, metabolic kinetics, HPLC-DAD-MS/MS, UFLC-MS/MS

## Abstract

Ganoderic acid A (GAA), a representative active triterpenoid from *Ganoderma lucidum*, has been reported to exhibit antinociceptive, antioxidative, cytotoxic, hepatoprotective and anticancer activities. The present study aims (1) to identify GAA metabolites, *in vivo* by analyzing the bile, plasma and urine after intravenous administration to rats (20 mg/kg), and *in vitro* by incubating with rat liver microsomes (RLMs) and human liver microsomes (HLMs); (2) to investigate the metabolic kinetics of main GAA metabolites. Using HPLC-DAD-MS/MS techniques, a total of 37 metabolites were tentatively characterized from *in vivo* samples based on their fragmentation behaviors. The metabolites detected in *in vitro* samples were similar to those found *in vivo*. GAA underwent extensive phase I and II metabolism. The main metabolic soft spots of GAA were 3, 7, 11, 15, 23-carbonyl groups (or hydroxyl groups) and 12, 20, 28 (29)-carbon atoms. Ganoderic acid C_2_ (GAC_2_) and 7β,15-dihydroxy-3,11,23-trioxo-lanost-26-oic acid were two main reduction metabolites of GAA, and their kinetics followed classical hyperbolic kinetics. The specific isoenzyme responsible for the biotransformation of the two metabolites in RLMs and HLMs was CYP3A. This is the first report on the comprehensive metabolism of GAA, as well as the metabolic kinetics of its main metabolites.

## Introduction

*Ganoderma lucidum* (GL), an ancient remedy, has been used for increasing energy, improving immunity, and promoting health and longevity for over 2,000 years in Asian countries, particularly China, Japan and Korea (Wang et al., 2007). GL is commonly used as a dietary supplement or prescription in clinics to cure many diseases. Polysaccharides and triterpenoids are its two main types of components and considered to be responsible for the most of its pharmacological activities (Cheng P.-G. et al., 2013). Triterpenoids are highly oxygenated lanostane. Up to now, more than 140 triterpenoids have been isolated from the fruiting bodies, spores and mycelia of GL (Guo X.-Y. et al., 2013). Ganoderic acid A (GAA), generally exists in Ganoderma genus, is one of the most abundant triterpenoids of GL, and can be viewed as a marker component for evaluating GL quality (Zhao et al., 2006; Lu et al., 2012). In a previous study, GAA was chosen as the single reference substance for multiple components determination for quantity control of GL (Da et al., 2015). GAA reportedly exhibited antinociceptive (Koyama et al., 1997), antioxidative (Zhu et al., 1999), cytotoxic (Guan et al., 2008) and hepatoprotective activities (Kim et al., 1999), especially anticancer activity (Jiang et al., 2008; Yao et al., 2012; Das et al., 2015; Radwan et al., 2015; Shao et al., 2015), which is the most attractive character of this compound.

Recently, the anticancer activity of GAA attached the considerable attention of scientists. Recent studies showed that GAA exhibits antitumor activity on human osteosarcoma (Shao et al., 2015), lymphoma (Radwan et al., 2015), meningioma (Das et al., 2015) and breast cancer cells (Jiang et al., 2008) through suppressing growth and invasive behavior and/or inducing apoptosis of cancer cells. GAA could also enhance chemosensitivity of HepG2 cells to Cisplatin (Yao et al., 2012). Radwan et al. firstly studied the anticancer activity of GAA *in vivo* and revealed that GAA can significantly prolong the survival of EL4 syngeneic mice and decrease tumor metastasis to the liver, and enhance cell-mediated immune responses by attenuating myeloid-derived suppressor cells (Radwan et al., 2015). Thus, GAA can be viewed as a promising anticancer candidate or used in combination with conventional chemotherapeutic agents for treatment of cancer.

So far, there is no any published research on the metabolism of GAA. This study aims to detect and identify GAA metabolites *in vivo* by analyzing the bile, plasma and urine after intravenous administration to rats and *in vitro* by incubating with rat liver microsomes (RLMs) and human liver microsomes (HLMs), analyzing by using a simple and accurate HPLC-DAD-MS/MS method. Besides, the metabolic kinetics of the main reduction product of GAA were determined by a sensitive and rapid UFLC-MS/MS method.

## Materials and methods

### Chemicals and reagents

GAA was purchased from Weikeqi Biotechnology Co, Ltd. (Chengdu, Sichuan province, China). Ganoderic acid B, ganoderic acid C_2_ (GAC_2_) and ganoderic acid D were kindly provided by professor Ruo-Yun Chen (Institute of Materia Medica, Chinese Academy of Medical Sciences and Peking Union Medical College, Beijing, China). Glycyrrhizic acid (GLA), α-naphthoflavone, quinidine, fluconazole, diethyldithiocarbamate and uridine 5′-diphosphoglucuronic acid (UDPGA) were obtained from Sigma-Aldrich Chemical Co. (St. Louis, MO, USA). Ticlopidine and ketoconazole were purchased from the National Institutes for Food and Drug Control (Beijing, China). β-nicotinamide adenine dinucleotide phosphate (NADPH) was purchased from Roche Molecular Biochemicals (Basel, Switzerland). A pooled Sprague Dawley rat liver microsomes (RLMs) was obtained from RILD Research Institute for Liver Diseases Co. Ltd (Shanghai, China). A pooled human liver microsomes (HLMs) was obtained from BD Gentest Corporation (Woburn, MA, USA). Methanol and ethyl acetate of HPLC grade, and acetonitrile and formic acid of LC/MS grade, were obtained from Fisher Co. Ltd. (Waltham, MA, USA). Milli-Q (Millford, MA, USA) water was used throughout the study.

### Animals

Male Sprague-Dawley rats (200 ± 20 g) were supplied by Beijing Vital Laboratory Animal Technology (Beijing, China). The animal experiment was approved by the Animal Ethics Committee at the Institute of Medicinal Plant Development, Chinese Academy of Medical Sciences. The rats were housed under standard conditions of temperature (23 ± 2°C), humidity (60 ± 5%) and light (12 h light-dark cycle) in a specific pathogen-free animal room, and had free access to standard rodent diet and water before the experiments. On the day before the experiment, the rats were suffered with light surgeries. Under anesthesia by an intraperitoneal dose of 10% chloral hydrate at 3.50 ml/kg, a polyethylene catheter (0.50 mm ID, 1.00 mm OD, Portex Limited, Hythe, Kent, England) was cannulated into the right jugular vein for intravenous drug administration and blood collection, and another catheter (0.28 mm ID, 0.61 mm OD, Portex Limited, Hythe, Kent, England) was cannulated into the bile fistul for bile collection. After surgery, the rats were placed individually in metabolism cages and allowed to recover for at least 24 h. The rats were fasted over night with free access to water prior to drug administration.

### Drug administration and sample preparation

GAA (25 mg) was dissolved in 500 μl of physiological saline containing 2% sodium carbonate and diluted with physiological saline to make the volume of 5 ml (Cheng C.-R. et al., 2013). The solution was given to rats (*n* = 5) intravenously at 20 mg/kg through the jugular vein catheter. After dosing and blood sample collection, 0.2 ml of normal saline with 20 units of heparin was injected into the body through the catheter to flush the catheter and prevent blood coagulation.

Rats were held in metabolism cages, blood was collected into heparinized tubes from the jugular vein before (blank) and at 5 and 30 min post dosing, and then centrifuged at 1,500 g for 10 min for plasma separation. At the same time, bile and urine samples were collected before (blank) and over the period of 0–4 h post dosing. All of the collected blank plasma, blank bile, blank urine, dosing plasma, dosing bile or dosing urine from five rats were pooled together respectively and stored at −20°C until assay.

For plasma, an aliquot of 200 μl sample was mixed with 20 μl HCl solution (1 M), and then extracted with 1.5 ml of ethyl acetate by vortex-mixing for 10 min. The ethyl acetate layer was collected and concentrated to dryness by a vacuum concentrator. The residue was reconstituted in 100 μl 80% methanol and centrifuged at 20,000 g for 15 min. The supernatant was collected for HPLC-DAD-MS/MS assay. For bile, an aliquot of 150 μl sample was mixed with 150 μl 80% methanol and 10 μl 5% formic acid, and then centrifuged at 20,000 g for 15 min. The supernatant was collected for HPLC-DAD-MS/MS assay. For urine, an aliquot of 2 ml sample was loaded on a pretreated ODS-C18 cartridge (Agilent AccuBOND, 500 mg). After washing with 5 ml of water, the cartridge was eluted with 10 ml of methanol. The methanol eluate was collected and concentrated to dryness by a vacuum concentrator at 37°C. The residue was reconstituted in 200 μl 80% methanol and centrifuged at 20,000 g for 15 min, and the supernatant was collected for HPLC-DAD-MS/MS assay.

### *In vitro* metabolite identification

For *in vitro* metabolites identification, the enzyme incubation was performed in a medium containing 1.0 mg protein /ml RLMs or HLMs, 100 μM of GAA, 3.3 mM of MgCl_2_, 3.0 mM of NADPH, 3.0 mM of UDPGA and 100 mM of sodium phosphate buffer (pH 7.4). The total volume was 500 μl. The metabolism reaction was initiated by adding NADPH and UDPGA after 10 min preincubation at 37°C. The reaction was maintained at 37°C for 1 h and terminated by adding with 500 μl of ice-cold acetonitrile. The mixture was centrifuged at 20,000 g for 15 min, and 10 μl of the supernatant was directly injected for HPLC-DAD-MS/MS assay. Control samples were prepared as described above by using inactivated enzymes in the incubation system. Each of the incubations was performed in duplicate.

### Formation kinetics of the metabolites M2 and M4

The formation kinetics of the two main reduction metabolites M2 (GAC_2_) and M4 (7β,15-dihydroxy-3,11,23-trioxo-lanost-26-oic acid) were determined. The incubations were performed in a medium (100 μl) containing 0.25 mg protein /ml RLMs or HLMs, different concentrations of GAA (1–50 μM), 3.3 mM of MgCl_2_, 3.0 mM of NADPH and 100 mM of sodium phosphate buffer (pH 7.4). After 10 min of preincubation, GAA at each concentration was incubated with RLMs and HLMs for 5 and 30 min, respectively. The reactions were terminated by adding with 100 μl of ice-cold acetonitrile containing 2 μM GLA used as internal standard (IS). After centrifugation, metabolites in the mixture were analyzed by UFLC-MS/MS detection as described below. The metabolite formation rates vs. GAA concentrations were plotted to obtain the Michaelis-Menten constant (K_m_) and maximum velocity (V_max_) values of the metabolic reactions. Each of the incubations was performed in triplicate.

### Participation of CYP enzymes in formation of the metabolites M2 and M4

GAA (10 μM) was incubated with 0.25 mg/ml RLMs or HLMs in the incubation system as described in Section Formation Kinetics of the Metabolites M2 and M4, in the present or absent of the following CYP enzymes inhibitors, 10 μM α- naphthoflavone (for CYP1A2), 100 μM ticlopidine (for CYP2C19), 10 μM quinidine (for CYP2D), 10 μM ketoconazole (for CYP3A), 100 μM fluconazole (for CYP2C9) and 100 μM diethyldithiocarbamate (for CYP2E1), respectively. The M2 and M4 formation rates in the present and absent of inhibitors were compared. The percentage of inhibition was calculated. Each of the incubations was performed in triplicate.

### HPLC-DAD-MS/MS conditions for metabolite identification

The identification of metabolites were conducted by a HPLC-DAD-MS/MS system equipped with an Agilent 1,260 HPLC system (Agilent Technologies, Santa Clara, CA, USA), 1,260 diode array detector (DAD) and 4,500 Q-Trap mass spectrometer with electrospray ionization source (AB SCIEX, Framingham, MA, USA). Chromatographic separation was performed on a C_18_ column (100 × 4.6 mm, 2.4 μm, BDS hypersil, Thermo, PA) maintained at 40°C. The mobile phase consisted of acetonitrile (A) and 0.1% formic acid aqueous solution (B) with following gradient elution at a flow rate of 0.40 ml/min: 0–10 min, 20–35% A; 10–20 min, 35% A; 20–25 min, 35–65% A; 25–30 min, 65% A; 30–35 min, 65–20% A; and 35–45 min, 20% A. The DAD spectral data was collected from 190 to 400 nm. The injection volume of all the tested samples was 10 μl. Mass data were acquired in negative mode under the following conditions, ion spray voltage −4,500 V, ion source temperature 450°C, curtain gas 10 psi, nebulizer gas 60 psi and auxiliary gas 60 psi. Data were collected using Analyst 1.6.2 (Applied Biosystems) in both first quadrupole (Q1) mass scan mode and multiple reaction monitoring (MRM) independent data acquisition (IDA) mode using enhanced product ion scans (EPI) and enhanced resolution (ER) in the ion trap mode.

### UFLC-MS/MS conditions for quantitation of the metabolites M2 and M4

An LC-MS/MS system consisted of a Shimadzu UFLC system (Kyoto, JPN) and an AB SCIEX 5500 Q-Trap mass spectrometer with electrospray ionization source (Foster City, CA, USA). The chromatographic separation was performed on a C_18_ column (50 × 2.1 mm, 1.7 μm, Syncronis aQ, Thermo, PA) maintained at 20°C. The mobile phase consisted of acetonitrile (A) and 0.1% formic acid aqueous solution (B) with following gradient elution at a flow rate of 0.30 ml/min: 0–2.50 min, 37% A; 2.50–2.75 min, 37–45% A; 2.75–3.75 min, 45% A; 3.75–4.00 min, 45–37% A; and 4.00–5.00 min, 37% A. Mass spectrometry was adopted in negative mode, with ion spray voltage −4,500 V, ion source temperature 550°C, curtain gas 10 psi, nebulizer gas 55 psi and auxiliary gas 35 psi. The quantification assay was carried out by using MRM mode with the precursor-product ion pairs of *m/z* 517.3/287.3, 517.3/287.3, 821.4/351.0 for M2, M4, and GLA, respectively. Declustering potentials were −240 and −32 V, entrance potentials were −10 and −23 V, collision energy were −50 and −58 V, and cell exit potential were −21 and −16 V for M2/M4 and GLA, respectively. The data acquisition and peak integration were performed using Analyst software (Version 1.6.2, AB SCIEX).

### Metabolic kinetic analysis

The kinetic parameters of GAA metabolism by RLMs or HLMs were calculated by fitting the data to the hyperbolic Michaelis-Menten model:

$$V=V_{\text{max}}\times \frac{\left[S\right]}{K_{\text{m}}+[S]}$$

Where *V*_max_ is the maximal velocity of formation, *S* is the concentration of substrate, *K*_m_ is the substrate concentration of half maximal velocity, and the intrinsic clearance (*CL*_int_) is calculated as *V*_max_/*K*_m_.

### Statistical analysis

The statistical difference was performed by Student's *t*-test, and *p* < 0.05 was considered statistically significant.

## Results and discussion

### Characterization of GAA metabolites in rats

**Figure 1:**
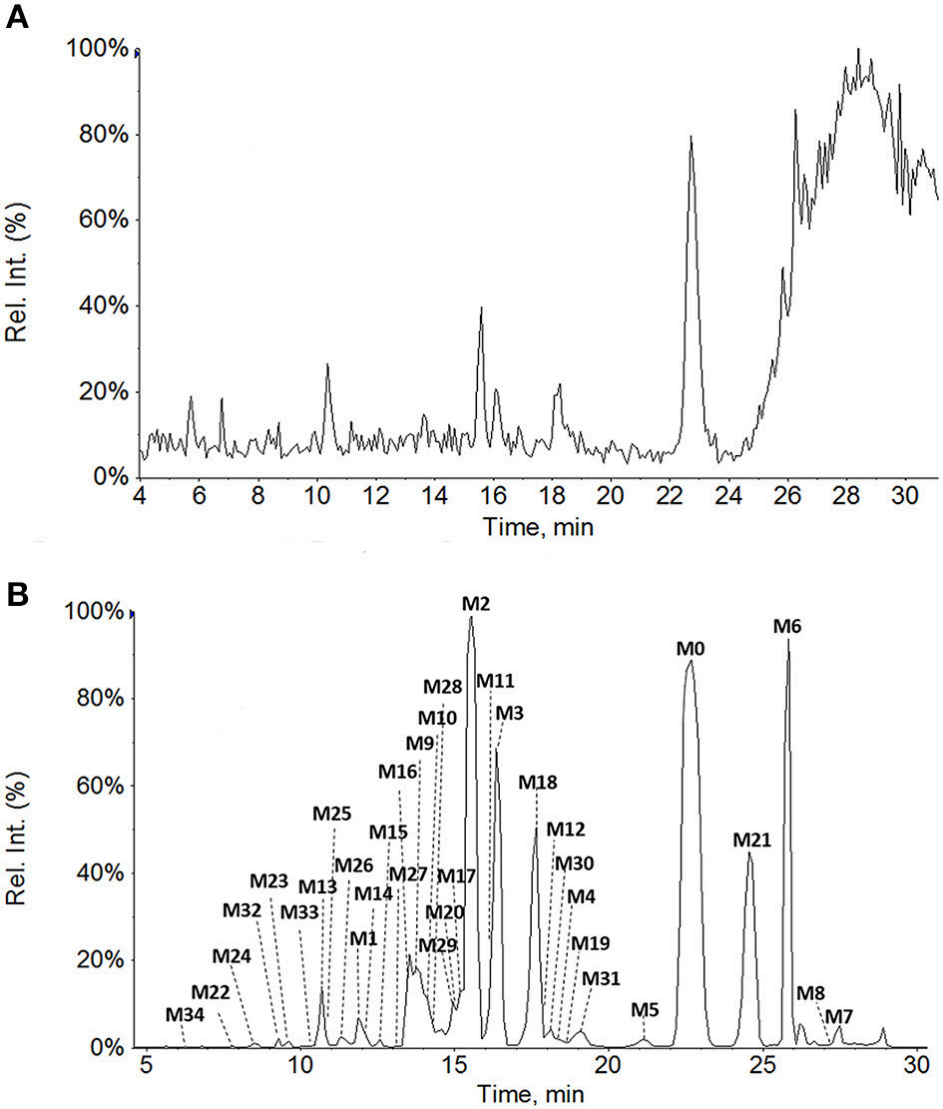
**Total ion chromatograms of rat bile samples before (A)** and after intravenous administration of GAA at 20 mg/kg **(B)**.

**Figure 2:**
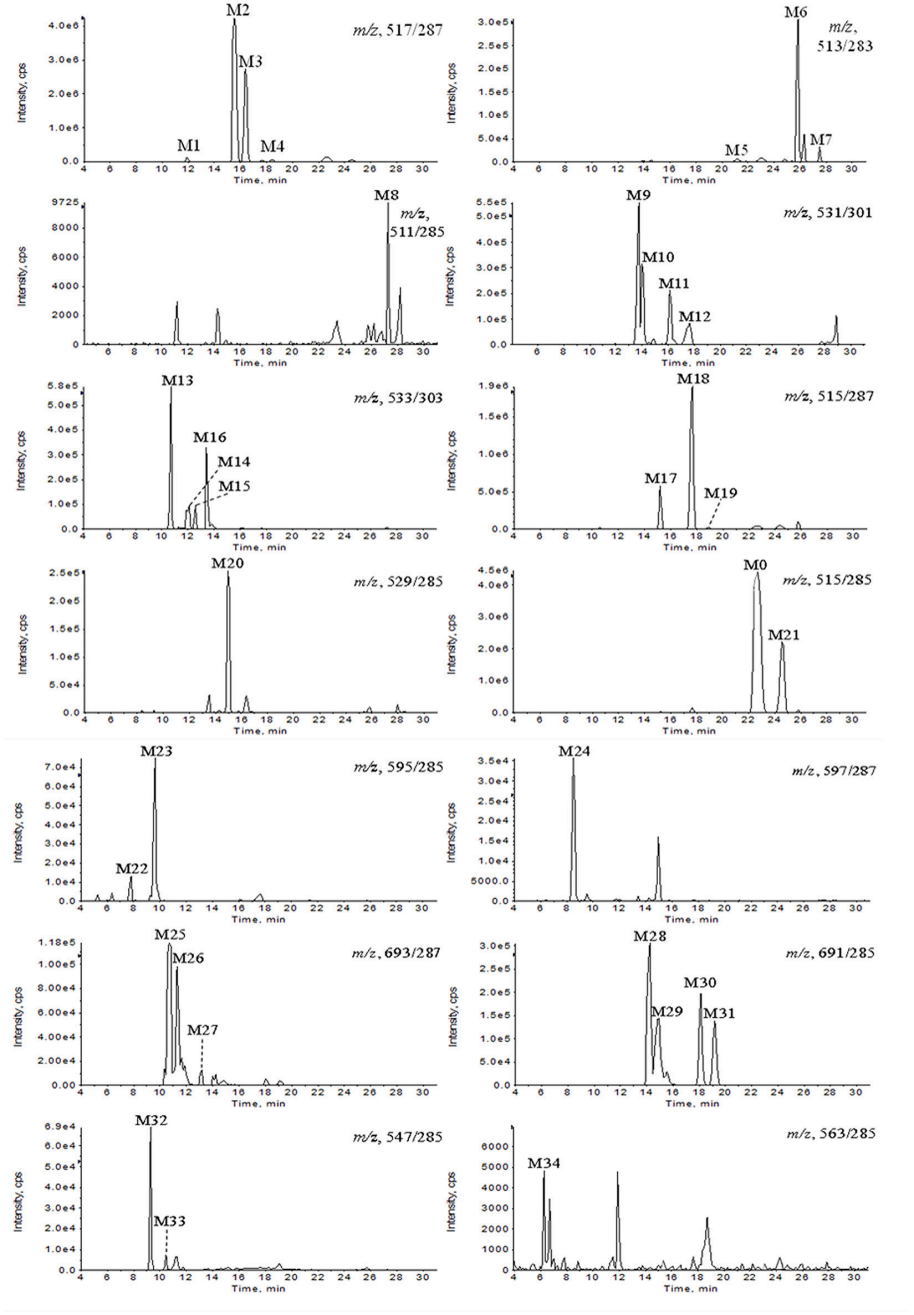
**Extracted ion chromatograms of GAA metabolites in rat bile sample after intravenous administration of GAA at 20 mg/kg**.

**Figure 3:**
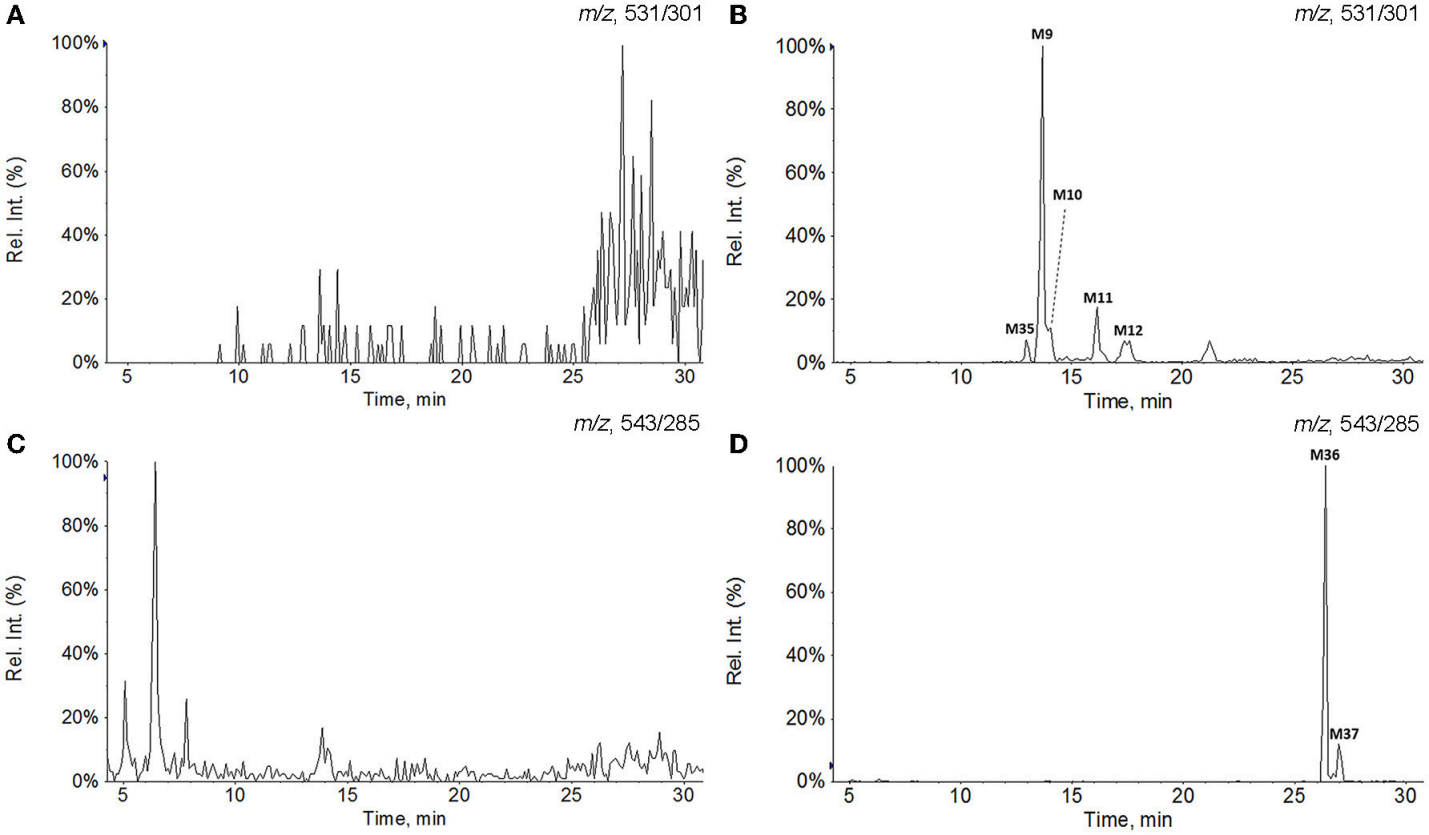
**Extracted ion chromatograms of GAA hydroxylation metabolites in plasma and GAA oxidoreduction metabolites in urine. (A)** Blank plasma; **(B)** plasma sample after intravenous administration of GAA at 20 mg/kg; **(C)** blank urine; **(D)** urine sample after intravenous administration of GAA at 20 mg/kg.

All of the biological samples were analyzed by HPLC-DAD-MS/MS for their retention times, UV spectra and mass fragmentation pathways. Comparing the chromatograms of MRM survey scan and Q1 full scan of the dosing samples with their corresponding blank samples, a total of 37 metabolites of GAA as well as parent GAA (M0) were detected and identified or tentatively characterized from the bile (34), plasma (22) and urine (12) samples. Table 1 lists the chromatographic, spectra and fragment ions data of GAA and its metabolites. Total ion chromatograms and extracted ion chromatograms of the tested samples are showed in Figures 1–3.

**Table 1:** **Characterization of GAA and its metabolites by HPLC-DAD-MS**.

**Metabolite**	**Identification**	**UV λ_max_ (nm)**	**t_R_ (min)**	**Formula**	***m/z***	**Fragment Ions**	**Detected resource**				
							**1**	**2**	**3**	**4**	**5**
M0	GAA	256	22.69	C_30_H_44_O_7_	515	515, 497, 479, 453, 435, 355, 301, 300, 299, 285, 283, 195	+	+	+	+	+
M1	3,7β,15,23-tetrahydroxy-11-oxo-lanost-8,24-dien-26-oic acid	246	11.89	C_30_H_46_O_7_	517	517, 499, 481, 455, 437, 303, 302, 301, 287, 195	+	−	−	−	−
M2	Ganoderic acid C2	258	15.54	C_30_H_46_O_7_	517	517, 499, 481, 455, 437, 303, 302, 301, 287, 195	+	+	+	+	+
M3	7β,11,15-trihydroxy-3,23-dioxo-lanost-8-en-26-oic acid	250	16.26	C_30_H_46_O_7_	517	517, 499, 481, 455, 437, 303, 302, 301, 287, 195	+	+	+	−	−
M4	7β,15-dihydroxy-3,11,23-trioxo-lanost-26-oic acid	246	18.43	C_30_H_46_O_7_	517	517, 499, 481, 455, 437, 303, 302, 301, 287, 195	+	+	−	−	+
M5	7β,23-dihydroxy-3,11,15-trioxo-lanost-8,24-dien-26-oic acid	246	21.13	C_30_H_42_O_7_	513	513, 495, 451, 301, 285, 283, 247, 149	+	+	+	−	−
M6	Ganoderic acid D	246	25.81	C_30_H_42_O_7_	513	513, 495, 451, 301, 285, 283, 247, 149	+	+	+	−	−
M7	Ganoderic acid J	250	27.47	C_30_H_42_O_7_	513	513, 495, 451, 436, 421, 399, 335, 301, 285, 283, 247	+	+	+	−	−
M8	Ganoderic acid E	250	27.26	C_30_H_40_O_7_	511	511, 493, 449, 301, 285, 247, 149	+	−	−	−	−
M9	12-hydroxy ganoderic acid A	252	13.74	C_30_H_44_O_8_	531	531, 513, 501, 495, 483, 465, 451, 317, 301, 195	+	+	−	+	+
M10	11,12,15-trihydroxy-3,7,23-trioxo-lanost-8-en-26-oic acid	246	13.99	C_30_H_44_O_8_	531	531, 513, 501, 495, 483, 465, 451, 319, 317, 303, 301	+	+	−	−	−
M11	20-hydroxy ganoderic acid A	242	16.15	C_30_H_44_O_8_	531	531, 513, 495, 469, 451, 301, 285, 193	+	+	−	+	+
M12	11,15,20-trihydroxy-3,7,23-trioxo-lanost-8-en-26-oic acid	246	17.63	C_30_H_44_O_8_	531	531, 513, 495, 469, 451, 303, 301, 286, 285, 249, 193	+	+	−	+	+
M13	3,7β,15,28(29)-tetrahydroxy-11, 23-dioxo-lanost-8-en-26-oic acid	246	10.69	C_30_H_46_O_8_	533	533, 515, 497, 453, 423, 317, 303, 195	+	+	−	+	−
M14	Ganoderic acid L	246	12.08	C_30_H_46_O_8_	533	533, 515, 497, 453, 423, 303, 301, 287, 193	+	+	−	−	−
M15	12-hydroxy ganoderic acid C_2_	242	12.46	C_30_H_46_O_8_	533	533, 515, 497, 485, 467, 453, 423, 319, 317, 303, 195	+	+	−	+	−
M16	7β,11,12,15-tetrahydroxy-3,23-dioxo-lanost-8-en-26-oic acid	254	13.42	C_30_H_46_O_8_	533	533, 515, 485, 467, 453, 319, 317, 303, 195	+	+	−	+	−
M17	3,7β,23-trihydroxy-11,15-dioxo-lanost-8,24-dien-26-oic acid	258	15.17	C_30_H_44_O_7_	515	515, 497, 453, 303, 301, 287, 249, 195	+	+	−	−	−
M18	Ganoderic acid B	246	17.65	C_30_H_44_O_7_	515	515, 497, 453, 303, 301, 287, 249, 195, 193, 149	+	+	−	−	−
M19	7β,11-dihydroxy-3,15,23-trioxo-lanost-8-en-26-oic acid	248	18.91	C_30_H_44_O_7_	515	515, 497, 453, 303, 287, 285, 249, 193	+	+	−	−	−
M20	12-hydroxy ganoderic acid D	246	14.96	C_30_H_42_O_8_	529	529, 511, 499, 481, 467, 317, 301, 287, 285, 193	+	+	−	−	−
M21	11,15-dihydroxy-3,7,23- trioxo-lanost-8-en-26-oic acid	254	24.22	C_30_H_44_O_7_	515	515, 497, 479, 453, 435, 405, 355, 303, 301, 300, 299, 286, 285, 195, 149	+	+	+	−	−
M22	15,23-dihydroxy-3,11-dioxo-lanost-8, 24-dien-26-oic acid-7β-O-sulfation	244	7.85	C_30_H_44_O_10_S	595	595, 515, 497, 479, 453, 435, 421, 405, 301, 300, 299, 285, 283, 195	+	−	−	−	−
M23	Ganoderic acid A 7-O-sulfation	242	9.80	C_30_H_44_O_10_S	595	595, 563, 515, 497, 479, 453, 435, 285, 269, 149	+	−	−	−	−
M24	Ganoderic acid C2 3-O-sulfation	254	8.51	C_30_H_46_O_10_S	597	597, 517, 499, 481, 455, 437, 303, 302, 301, 287	+	−	−	−	−
M25	Ganoderic acid C2 3-O-glucuronide	246	10.73	C_36_H_54_O_13_	693	693, 517, 499, 481, 455, 437, 303, 302, 301, 287, 235, 195	+	−	−	+	−
M26	Ganoderic acid C2 7-O-glucuronide	242	11.31	C_36_H_54_O_13_	693	693, 517, 499, 481, 455, 437, 303, 302, 301, 287, 235, 195	+	−	−	−	−
M27	Ganoderic acid C2 15-O-glucuronide	246	13.13	C_36_H_54_O_13_	693	693, 515, 499, 481, 455, 437, 303, 302, 301, 287, 235, 195	+	−	−	−	−
M28	Ganoderic acid A 7-O-glucuronide	248	14.20	C_36_H_52_O_13_	691	691, 515, 497, 479, 453, 435, 355, 301, 300 299, 285, 233, 195, 175	+	+	+	+	+
M29	15-hydroxy-3,7,23-trioxo-lanost-8-en-26-oic acid-11-O-glucuronide	246	14.82	C_36_H_52_O_13_	691	691, 515, 497, 479, 453, 435, 355, 301, 300, 299, 287, 285, 233, 195, 175	+	+	+	−	−
M30	Ganoderic acid A 15-O-glucuronide	246	18.10	C_36_H_52_O_13_	691	691, 513, 495, 477, 451, 435, 355, 301, 300, 299, 285, 233, 195, 177	+	−	+	−	−
M31	11-hydroxy-3,7,23-trioxo-lanost-8-en-26-oic acid-15-O-glucuronide	246	19.17	C_36_H_52_O_13_	691	691, 513, 495, 477, 451, 435, 355, 301, 300, 299, 287, 285, 233, 195, 177	+	−	+	−	−
M32	12,28(29)-dihydroxy ganoderic acid A	244	9.28	C_30_H_44_O_9_	547	547, 529, 517, 511, 499, 481, 303, 287, 285, 195	+	−	−	−	−
M33	12,20-dihydroxy ganoderic acid A	242	10.46	C_30_H_44_O_9_	547	547, 529, 517, 511, 499, 481, 317, 301, 287, 285, 211, 193	+	−	−	−	−
M34	12,20,28 (29)-trihydroxy ganoderic acid A	244	6.25	C_30_H_44_O_10_	563	563, 545, 533, 527, 515, 483, 303, 287, 285, 193	+	−	−	−	−
M35	28(29)-hydroxy ganoderic acid A	250	12.97	C_30_H_44_O_8_	531	531, 513, 495, 451, 317, 315, 301, 195	+	+	−	−	+
M36	12,28(29)-dicarbonyl ganoderic acid A	232	26.30	C_31_H_44_O_8_	543	543, 525, 515, 497, 481, 479, 463, 453, 435, 355, 301, 300, 299, 285, 195	+	−	+	−	−
M37	11,15-dihydroxy-3,7,12,23,28(29)-pentaoxo-lanost-8-en-26-oic acid	230	26.98	C_31_H_44_O_8_	543	543, 525, 515, 497, 481, 479, 463, 453, 435, 355, 303, 301, 300, 299, 286, 285, 195	+	−	+	−	−

*1, rat bile; 2, rat plasma; 3, rat urine; 4, RLMs; 5, HLMs*.

*+, detected; –, not detected*.

**Figure 4:**
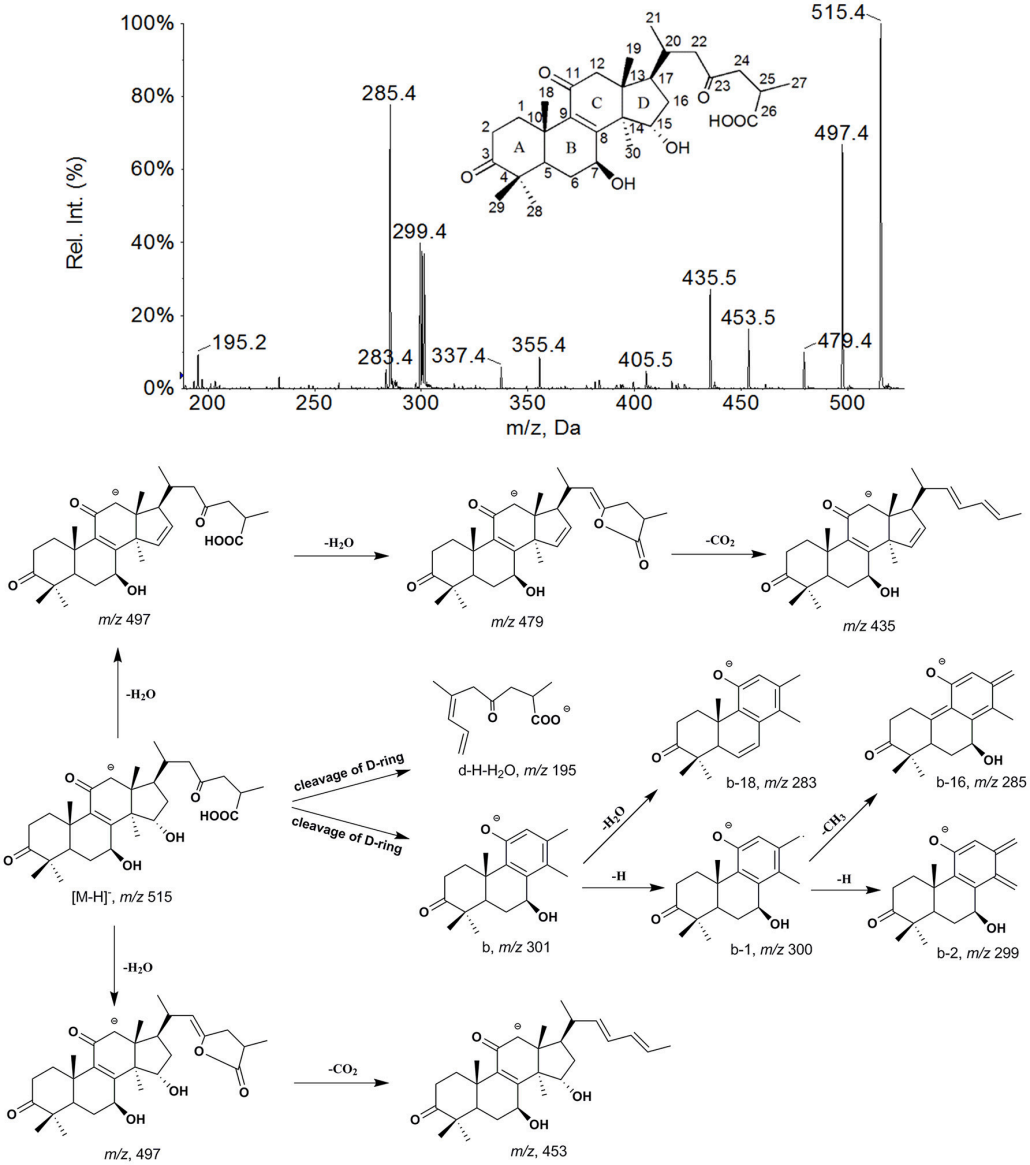
**The mass spectrum and proposed fragmentation pathways of GAA**.

As shown in Figure 4, the [M−H]^−^ ion *m/z* 515 of M0 mainly generated the fragment ions at *m/z* 497, 479, 453, and 435 by neutral losses of H_2_O and CO_2_, and produced characteristic fragment ions of 301 (b), 300 (b−1), 299 (b−2), 285 (b−H−CH_3_), 283 (b−H_2_O) and 195 (d−H−H_2_O) by cleavage of D-ring. The metabolites were identified or tentatively characterized based on their fragmentation pathways (Supplementary Figures 1–6) and comparison with the parent compound.

The metabolites M1 (retention time *t*_*R*_ = 11.89 min), M2 (*t*_*R*_ = 15.54 min), M3 (*t*_*R*_ = 16.26 min), and M4 (*t*_*R*_ = 18.43 min) all showed a same [M−H]^−^ ion at *m/z* 517 (Table 1) with different retention times. Their fragmentation were very similar to that of GAA and had the product ions at *m/z* 303 (b), 302 (b−1), 301 (b−2) and 287 (b−H−CH_3_), all 2 Da heavier than those of GAA, which were generated from ring D cleavage. This indicates that the reduction occurred on the A-, B- or C- rings of M0, that might be 3-carbonyl, 11-carbonyl or double bond between C-8 and C-9. Among these metabolites, M2 was found to be the most abundant *in vivo* (based on the chromatogram by DAD detector) and identified as GAC_2_ by comparing its retention time, UV spectrum and EPI spectrum with those of GAC_2_ authentic standard in HPLC-DAD-MS/MS experiments. With shorter retention time than that of M2, M1 could be inferred as a M2 reduction product, formed by that the 23-carbonyl group in the side chain of M2 was reduced into a hydroxyl group and a double bond was simultaneously formed between C-24 and C-25 (Guo X. et al., 2013). Therefore, M1 was identified as 3,7β,15,23-tetrahydroxy-11-oxo-lanost-8,24-dien-26-oic acid. With longer retention times, M3 and M4 might be inferred to be reduction products of GAA, at 11-carbonyl and double bond between C-8 and C-9, respectively, and named 7β,11,15-trihydroxy-3,23-dioxo-lanost-8-en-26-oic acid and 7β,15-dihydroxy-3,11,23-trioxo-lanost-26-oic acid, respectively, which are reported for the first time. In addition, the peak response of the metabolites with side chain modification was found lower than that of the corresponding metabolites without side chain modification, such as M1 < M2, M5 < M6, M17 < M18, and M22 < M23. It seems that the metabolites with side chain modification were more unstable than the metabolites without side chain modification. The peak response of M3 was higher than that of M4, therefore, M3 might not be side chain modification metabolite of M4.

Metabolites M5, M6, and M7 exhibited a same [M−H]^−^ ion at *m/z* 513, 2 Da lower than the parent GAA. It suggests that they were all dehydrogenation metabolites of GAA and the dehydrogenation could occur at the position of 7- or 15- OH. Metabolite M6 generated *m/z* 495 and 451 owing to the neutral loss of H_2_O and CO_2_ from the [M−H]^−^ ion, and *m/z* 247 owing to the cleavage of C-ring and a group ions of *m/z* 301 (b), 283 (b−H_2_O), and 285 (b−H−CH_3_), owing to the cleavage of D-ring. These results indicate that M6 might be a 7-hydroxy-15-oxo derivatives (Yang et al., 2007; Cheng et al., 2012). Compared with the authentic standard, M6 was identified as ganoderic acid D, which is the 15-OH dehydrogenation metabolite of GAA. With the similar mass spectra data, M5 (t_*R*_ = 21.13 min) exhibited shorter retention time than M6 (*t*_*R*_ = 25.81 min). Therefore, M5 was inferred to be 7β,23-dihydroxy-3,11,15-trioxo-lanost-8,24-dien-26-oic acid. M7 showed lower polarity with longer retention time compared to M6, suggesting that the dehydrogenation only could occur at 7- OH. M7 produced abundant ions at *m/z* 451, 436, and 421 originating from neutral loss of H_2_O, CO_2_ and CH_3_ from [M−H]^−^, as well as minor fragment ions at 301 (b+2), 285 (b−H−CH_3_+2) and 247 (a+2). The product ions of M7 generated from rings cleavage should be at 299 (b), 283 (b−H−CH_3_) and 245 (a), well, the fragment ions were actually at 301 (b+2), 285 (b−H−CH_3_+2) and 247 (a+2) for transference of two hydrogen atoms. The fragmentation pathway of M7 was in accordance with 7-carbonyl derivatives (Yang et al., 2007), therefore, M7 was identified as 15-hydroxy-3,7,11,23-tetraoxo-lanost-8-en-26-oic acid, i.e., ganoderic acid J.

Metabolite M8 gave the [M−H]^−^ ion at *m/z* 511 with the greatest intensity, which produced the fragment ions only 2 Da lower than those of M7. Therefore, M8 was identified as 3,7,11,15,23-pentaoxo-lanost-8-en-26-oic acid, i.e., ganoderic acid E (Yang et al., 2007; Guo X. et al., 2013).

Metabolites M17 (*t*_*R*_ = 15.17 min), M18 (*t*_*R*_ = 17.65 min), and M19 (*t*_*R*_ = 18.91 min) gave the same [M−H]^−^ ions at *m/z* 515, and produced the characteristic fragment ions at 303 and 249 owing to the cleavage of D-ring and C-ring, respectively, 2 Da heavier than those of M6. The fragmentation pathways of M17, M18, and M19 were very similar to that of M6, suggesting that their structures belonged to 7-hydroxy-15-oxo derivatives. Compared with the authentic standard, M18 was identified as ganoderic acid B. With shorter retention time than that of M18, M17 was inferred as M18 isomer at C-17 of side chain, and named 3,7β,23-trihydroxy-11,15-dioxo-lanost-8,24-dien-26-oic acid. With longer retention time than that of M18, M19 was inferred as 7β,11-dihydroxy-3,15,23-trioxo-lanost-8-en-26-oic acid.

M20 yielded the [M−H]^−^ ion at *m/z* 529, and its corresponding D-ring cleavage fragment ions (b, b−H−CH_3_) were 16 Da higher than those of M6, suggesting that M20 occurred hydroxylation reaction on A-, B-, or C- rings. The most dominant ions at *m/z* 499 ([M−H−CH_2_O]^−^) and 481 ([M−H−H_2_O−CH_2_O]^−^) in EPI spectrum of M20 resulted from a neutral loss of CH_2_O (−30 Da) from the ions at *m/z* 529 ([M−H]^−^) and 511 ([M−H−H_2_O]^−^), indicating that an additional hydroxyl group existed at C-12 of M20. Therefore, M20 was characterized as 12-hydroxy ganoderic acid D. M20 yielded fragment ion at *m/z* 193 (d−H−H_2_O) produced by D-ring cleavage, which also might be 195 (d−H−H_2_O+2) for the transference of two hydrogen atoms by 15-carbonyl. It is reported that fragment ion at *m/z* 193 indicated metabolites possessed a hydroxyl group at C-20 (Guo et al., 2012). Well, in the structure identification of M20, the 193 fragment ion was not a signature fragment for hydroxylation at C-20. Only when the fragment signals (b, b−H−CH_3_, and so on) resulted from D-ring cleavage of hydroxylation metabolites were similar to those of parent compound, suggesting that they possessed the same structure of A-, B- and C- rings as that of parent compound with no hydroxylation reaction, the 193 fragment would be a characteristic fragment ion indicating an extra hydroxyl group at C-20.

M21 showed a [M−H]^−^ ion at *m/z* 515, with similar fragmentation pathway to that of M0. After the prominent loss of H_2_O and CO_2_, mass fragmentation occurred on the ring skeleton, generating the ions at *m/z* 301, 300, 299, and 285. Minor ions at 303 (b+2) and 286 (b+1−H−CH_3_) were also observed, indicating that C-7 of M21 was oxidized to form a carbonyl group. In addition, M21 showed longer retention time (24.22 min) than that of M0 (22.69 min), indicating that M21 wasn't reduced at C-3. Therefore, M21 was identified as 11,15-dihydroxy-3,7,23-trioxo-lanost-8-en-26-oic acid.

The EPI spectra of M9, M10, M11, M12, and M35 showed their [M−H]^−^ ions all at *m/z* 531. The major D-ring cleavage fragment ions (b, b−H−CH_3_, and so on) of M9 and M10 were 16 Da higher than those of M0 and M21, respectively, indicating that hydroxylation reaction occurred on A-, B-, or C- rings. In MS spectra of M9 and M10, the neutral loss of CH_2_O (30 Da) from ions at *m/z* 531 ([M−H]^−^) and 513 ([M−H−H_2_O]^−^) yielded the dominant fragment ions at *m/z* 501 and 483, respectively, suggesting the presence of 12-OH (Yang et al., 2007; Guo X.-Y. et al., 2013). Therefore, M9 and M10 were characterized as 12-hydroxy ganoderic acid A and 11,12,15- trihydroxy-3,7,23-trioxo-lanost-8-en-26-oic acid, respectively. M35 produced similar major D-ring cleavage fragment ions with those of M9 except the absence of ions at *m/z* 501 and 483, suggesting that M35 occurred hydroxylation reaction on A-, B-, or C- rings while lacked 12-OH. Since the angular methyl group easily undergo hydroxylation reaction (Yang et al., 2007; Cheng et al., 2012; Guo X. et al., 2013), M35 was proposed as 28 (29)-hydroxy ganoderic acid A. The fragment signals resulted from D-ring cleavage of M11 and M12 were similar to those of M0 and M21, respectively, suggesting that they possessed the same structure of A-, B-, and C- rings as that of M0 and M21, accordingly. The spectra showed characteristic fragment ion at *m/z* 193 (d−H−H_2_O−H_2_O), indicating that M11 and M12 possessed an extra hydroxyl group at C-20 (Guo et al., 2012). Therefore, M11 and M12 were inferred as 20-hydroxy ganoderic acid A and 11,15,20-trihydroxy-3,7,23-trioxo-lanost-8-en-26-oic acid, respectively.

M13, M14, M15, and M16 gave the same [M−H]^−^ ions at *m/z* 533. M14 showed fragment ions at *m/z* 303 (b), 301 (b−2) and 287 (b−16), suggesting that it possessed the same structure of A-, B-, and C- rings as that of M2. Characteristic fragment ion at *m/z* 193 was observed, indicating the presence of 20-OH. Therefore, M14 was inferred as 20-hydroxy ganoderic acid C_2_. The major D-ring cleavage fragment ions (*m/z* 319, 317, and 303) of M15 were 16 Da higher than those of M2, indicating that hydroxylation reaction occurred on A-, B-, or C- rings. In EPI spectrum of M15, the key fragment ions at *m/z* 485 and 467, owing to a neutral loss of CH_2_O (30 Da) from the ions at *m/z* 515 ([M−H−H_2_O]^−^) and 497 ([M−H−H_2_O−H_2_O]^−^), respectively, were detected, indicating the presence of 12-OH. Therefore, M15 was identified as 12-hydroxy ganoderic acid C_2_. M13 produced similar fragment ions with those of M15 except the absence of ions at *m/z* 485 and 467, suggesting M13 lacked 12-OH. In its spectrum, the characteristic product ion at *m/z* 195 indicated that M13 possessed the same side chain as that of M2. According to that the angular methyl groups easily undergo hydroxylation reactions, M13 was tentatively identified as 3,7β,15,28(29)-tetrahydroxy-11,23-dioxo-lanost-8-en-26-oic acid. The fragmentation pathway of M16 was very similar to that of M15 with the fragment ions at *m/z* 485 ([M−H−H_2_O−CH_2_O]^−^), 467 ([M−H−H_2_O−H_2_O−CH_2_O]^−^), 317 (b−2) and 303 (b−H−CH_3_), while, retention time of M16 (*t*_*R*_ = 13.42 min) was longer than that of M15 (*t*_*R*_ = 12.46 min). Thus, M16 was tentatively identified as 7β,11,12, 15-tetrahydroxy-3, 23-dioxo-lanost-8-en-26-oic acid.

M23 had a [M−H]^−^ ion at *m/z* 595, and produced abundant [M−H−SO_3_]^−^ ion at *m/z* 515 by the neutral loss of 80 Da and other major fragment ions, which were very similar with those of M0. This suggests that it was sulfation of M0, and the sulfuric acid moiety could be in conjunction with 7- or 15- OH. Ganoderic acid A 7-O-sulfation had stronger polarity than ganoderic acid A 15-O-sulfation for relatively weak intramolecular hydrogen bonds, and could be easily generated. Therefore, M23 was tentatively identified as ganoderic acid A 7-O- sulfation. In the same way, M24 (*m/z*, 597) was inferred as ganoderic acid C_2_ 3-O-sulfation. The major fragment ions of M22 (*m/z*, 595) were the same as M23. While, its polarity was even stronger than M24 (*t*_*R*_ = 8.51 min), with retention time being 7.85 min, indicating that the difference in structure between M22 and M23 was on the side chain. Therefore, M22 was inferred as 15,23-dihydroxy-3,11-dioxo-lanost-8,24-dien-26-oic acid-7β-O-sulfation.

The EPI spectra of M25, M26, and M27 showed the same [M−H]^−^ ion at *m/z* 693, which produced abundant [M−H−GlcA]^−^ ion at *m/z* 517 by neutral loss of 176 Da and their other characteristic fragment ions were similar with those of GAC_2_. This suggested that they were monoglucuronide of GAC_2_ and the glucuronic acid moiety could conjunct with 3-, 7-, or 15- OH, respectively. The glucuronic acid moiety at 15-OH of GAC_2_ could form stronger intramolecular hydrogen bond than that at 7-OH, which would lower their polarities than that of ganoderic acid C_2_ 3-O-glucuronide. Therefore, M25, M26, and M27 were tentatively characterized as ganoderic acid C_2_ 3-O-glucuronide, ganoderic acid C_2_ 7-O-glucuronide and ganoderic acid C_2_ 15-O-glucuronide, respectively (Guo X.-Y. et al., 2013), in combination of their retention time.

The EPI spectra of M28, M29, M30, and M31 showed [M−H]^−^ ion at 691. By comparing the similarity of their major fragment ions with those of M0 and M21, it suggested that M28 and M30 were monoglucuronide of M0, and M29 and M31 were monoglucuronide of M21. Therefore, M28, M29, M30, and M31 were inferred to be ganoderic acid A 7-O-glucuronide, 15-hydroxy-3,7,23-trioxo-lanost-8-en-26-oic acid-11-O-glucuronide, ganoderic acid A 15-O-glucuronide and 11-hydroxy-3,7,23-trioxo-lanost-8-en-26-oic acid-15-O-glucuronide, respectively, dependent on their polarities.

M32 and M33 gave [M−H]^−^ ions both at *m/z* 547, and their fragmentation pathways were very similar to that of M0, suggesting that they were dihydroxylation of GAA. In EPI spectrum of M32 and M33, the neutral loss of CH_2_O (30 Da) from ions at *m/z* 547 ([M−H]^−^) and 529 ([M−H−H_2_O]^−^) yielded the dominant fragment ions at *m/z* 517 and 499, respectively, suggesting the presence of 12-OH. The key fragment ions at *m/z* 303 (b−CH_2_O) and 287 (b−H−CH_3_−CH_2_O) generated from the D-ring cleavage and neutral loss of CH_2_O in the spectrum of M32, meaning the b and b−H−CH_3_ being 333 and 317, respectively, indicated that the other hydroxylation occurred on an angular methyl group of ring A, C-28 or C-29. Therefore, M32 was inferred as 12, 28(29)-dihydroxy ganoderic acid A. In the spectrum of M33, the major D-ring cleavage fragment ions (*m/z* 317 and 301) were 16 Da higher than those of M0, indicating that only one hydroxylation reaction occurred on A-, B-, or C- rings, in addition, characteristic fragment ions at *m/z* 211 (d−H−H_2_O) and 193 (d−H−H_2_O−H_2_O) were observed, indicating the presence of 20-OH. Thus, M33 was inferred as 12, 20-dihydroxy ganoderic acid A. M34 (*m/z*, 563) was trihydroxylation of ganoderic acid A for the same fragmentation pathway as M0. M34 exhibited ions at *m/z* 533 ([M−H−CH_2_O]^−^), 515 ([M−H−H_2_O−CH_2_O]^−^), 303 (b−CH_2_O), 287 (b−H−CH_3_−CH_2_O) and 193 (d−H−H_2_O−H_2_O), indicating the presence of 12, 20, 28 (29)-OH. Thus, M34 was tentatively characterized as 12, 20, 28 (29)-trihydroxy ganoderic acid A.

The EPI spectra of M36 and M37 showed [M−H]^−^ ions at *m/z* 534, which produced abundant [M−H−CO]^−^ ions at *m/z* 515 originating from neutral losses of 28 Da, suggesting the presence of 12-carbonyl. The [M−H−CO]^−^ ions of M36 and M37 showed the same fragmentation patterns as those of M0 and M21, respectively. The key fragment ions at *m/z* 301 (b−CO) and 285 (b−H−CH_3_−CO) generated from the D-ring cleavage and neutral loss of CO in the spectrum of M36, meaning the b and b−H−CH_3_ being 329 and 313, respectively, indicated that another carbonyl on an angular methyl group of ring A, C-28 or C-29. Therefore, M36 was inferred to be 12,28(29)-dicarbonyl ganoderic acid A. In the same way, M37 was inferred to be 11,15-dihydroxy-3,7,12,23,28(29)-pentaoxo-lanost-8-en-26-oic acid. The –CH = O of M36 and M37 might be formed from M0 and M21, respectively, which firstly suffered hydroxylation at C-12 and C-28(29), and then dehydrogenation to form two carbonyls.

### Characterization of GAA metabolites produced by RLMs and HLMs

The metabolites found in RLMs incubations were similar with those detected in the bile after *i.v*. dose. Except for the monohydroxylated metabolites M9 and M11, the phase II metabolite M28 formed by glucuronidation was also found. The metabolite M2 was the most abundant metabolite in RLMs incubations, therefore, the relevant M2 metabolites were detected, including two mono-hydroxylated derivatives (M13 and M15) and one glucuronide conjugated derivative (M25).

The metabolites detected in HLMs incubations were fewer than those found in RLMs incubations, including two reduced metabolites (M2 and M4), three monohydroxylated metabolites (M9, M11, and M35) and one glucuronide conjugated metabolite (M28). Both M2 and M4 were abundant in HLMs incubations. However, M4 was very minor in rats and undetected in RLMs incubations.

### Proposed GAA metabolic pathway

By using HPLC-DAD-MS/MS techniques, a total of 37 metabolites were identified or tentatively characterized from the bile (34), plasma (22) and urine (12) samples of rats after *i.v*. dose of GAA. The results reveal that hepatocyte metabolism is the major route of clearance for GAA. The metabolic pathways of the in *vitro* samples were in consistent with those of the *in vivo* samples for rats. Nine metabolites (Table 1) were detected in RLMs reaction medium. The metabolites detected in RLMs were also found in the *in vivo* samples, indicating the metabolism consistency of GAA *in vitro* and *in vivo* for rats. For predicting GAA metabolism in humans and understanding the enzymes involved in drug metabolism differing between humans and rats, the identification of metabolites in HLMs was conducted. The GAA metabolites generated in HLMs were also observed in those *in vitro* and *in vivo* samples from rats, indicating the presence of the similar pathway of GAA metabolism in humans and rats.

**Figure 5:**
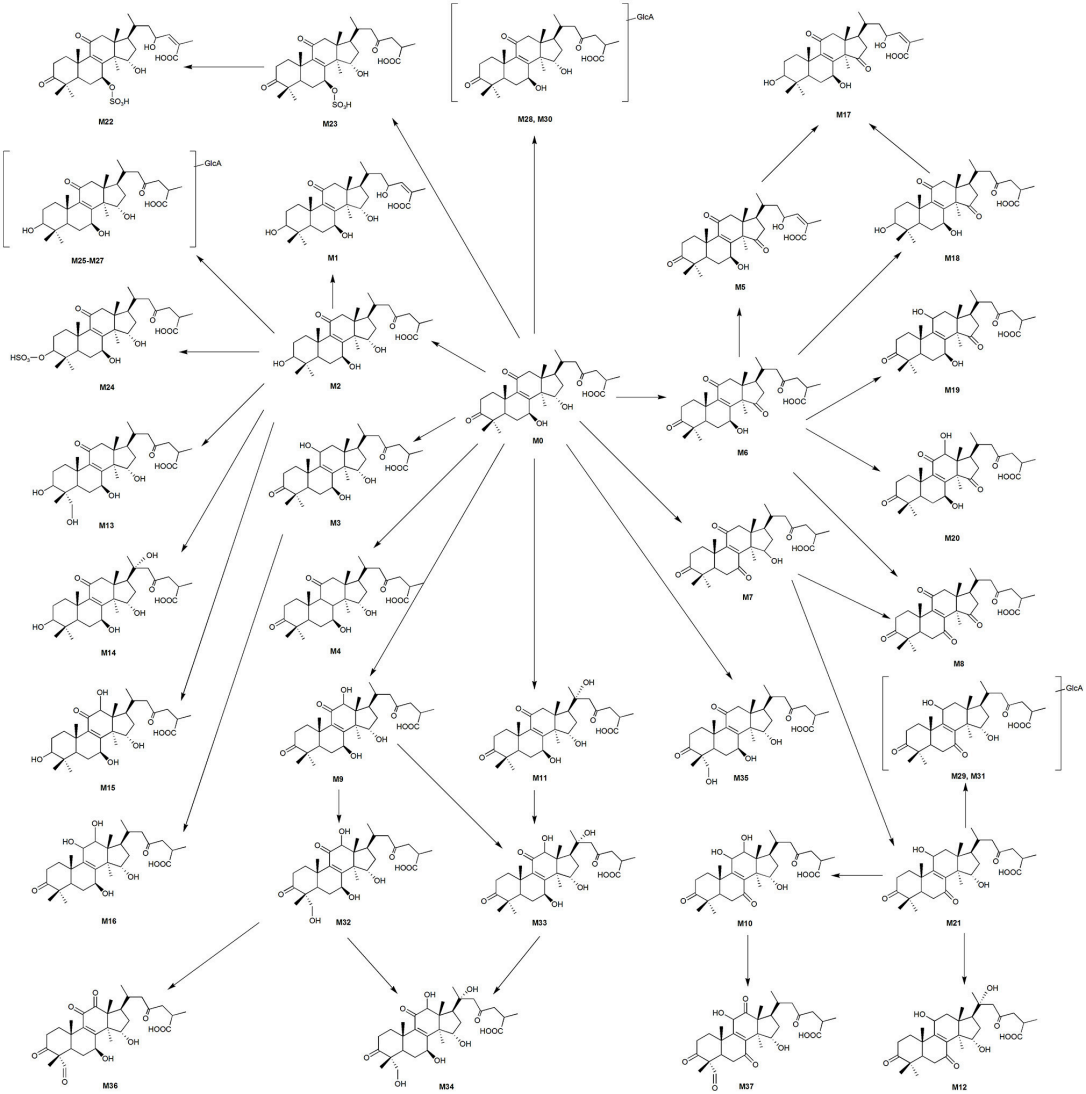
**Proposed metabolic pathways of GAA in rats and structures of its metabolites**.

Proposed metabolic pathways of GAA are showed in Figure 5. Both phase I and phase II metabolites were observed. The phase I metabolism in rats involved reduction, oxidation, oxidoreduction and hydroxylation. The reduction product, ganoderic acid C_2_, was the most abundant. Extensive hydroxylation products of GAA or its reduction/oxidation metabolites were observed in the bile and plasma. The phase II biotransformation observed included glucuronidation, and sulfation. There were a group of glucuronidation and sulfation metabolites of parent GAA and its reduction/oxidation metabolites in bile, and abundant glucuronidation metabolites of GAA and its reduction/oxidation metabolite in urine. Few glucuronidation metabolites were detected in plasma. These data indicated that the phase II metabolites were primarily excreted into the bile and urine.

GAA is composed of a highly oxygenated tetracyclic ring skeleton and an acidic side chain. The hydroxyl groups at C-3, C-7, and C-15 in GAA and its metabolites could be oxidized into carbonyl groups or conjugated with glucuronic acid and sulfuric acid. The carbonyl groups at C-3 and C-11 could be reduced to hydroxyl groups. The C-12, C-20, and C-28 (29) of GAA and its oxidoreduction metabolites were easily hydroxylated. The side chains at C-17 of GAA and its metabolites could be isomerized. Therefore, the main metabolic soft spots in the chemical structure of GAA were the 3, 7, 11, 15, 23- carbonyl groups (or hydroxyl groups) and 12, 20, 28 (29)- carbon atoms.

### Formation kinetics of the metabolites M2 and M4

**Figure 6:**
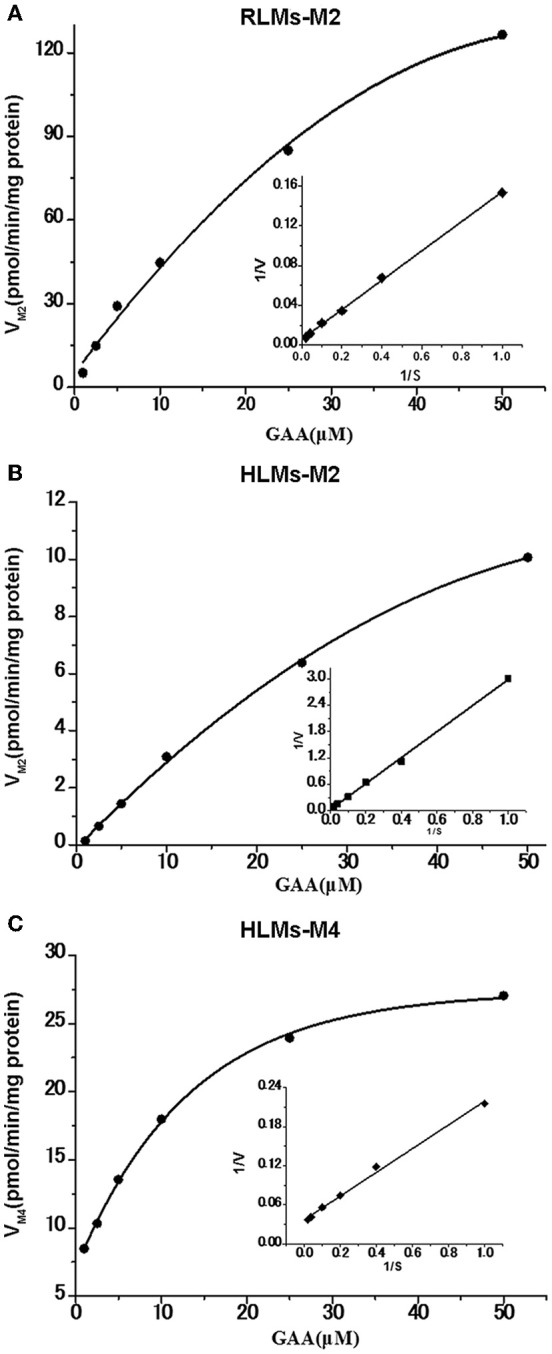
**Formation kinetics of GAA metabolites M2 and M4 in pooled RLMs (A)** or HLMs **(B,C)**. (mean ± SD, *n* = 3).

The reduction metabolites were found to be the most abundant both *in vivo* and *in vitro*. Their formation kinetics in RLMs and HLMs were examined to further explore the differences in reduction metabolism between RLMs and HLMs. The major reduction metabolite was M2 in RLMs, whereas M2 and M4 in HLMs. The kinetics of both M2 and M4 in RLMs and HLMs were fitted to the Michaelis-Menten equation. The kinetic parameters of M2 in RLMs were estimated as K_m_ of 28.80 μM, V_max_ of 185.81 pmol/min/mg, and Cl_int_ of 6.45 μl/min/mg. The kinetic parameters of M2 and M4 in HLMs were estimated as K_m_ of 91.81 and 6.12 μM, V_max_ of 30.18 and 29.70 pmol/min/mg, and Cl_int_ of 0.33 and 4.85 μl/min/mg, respectively. The Lineweaver-Burk transformation revealed a monophasic plot (Figure 6). The K_m_ value of M2 generated by HLMs was approximately 3-fold higher than that by RLMs. The V_max_ and CL_int_ values of M2 formation by RLMs were 6-fold and 20-fold higher than those by HLMs, respectively, suggesting that M2 was formated faster in RLMs than in HLMs. In HLMs, The K_m_ value of M2 was 15-fold higher than that of M4. The V_max_ values of M2 and M4 were similar. The CL_int_ of M4 formation was 15-fold higher than that of M2, suggesting that M4 was formated faster than M2 in HLMs.

### Participation of CYP enzymes in formation of the metabolites M2 and M4

**Figure 7:**
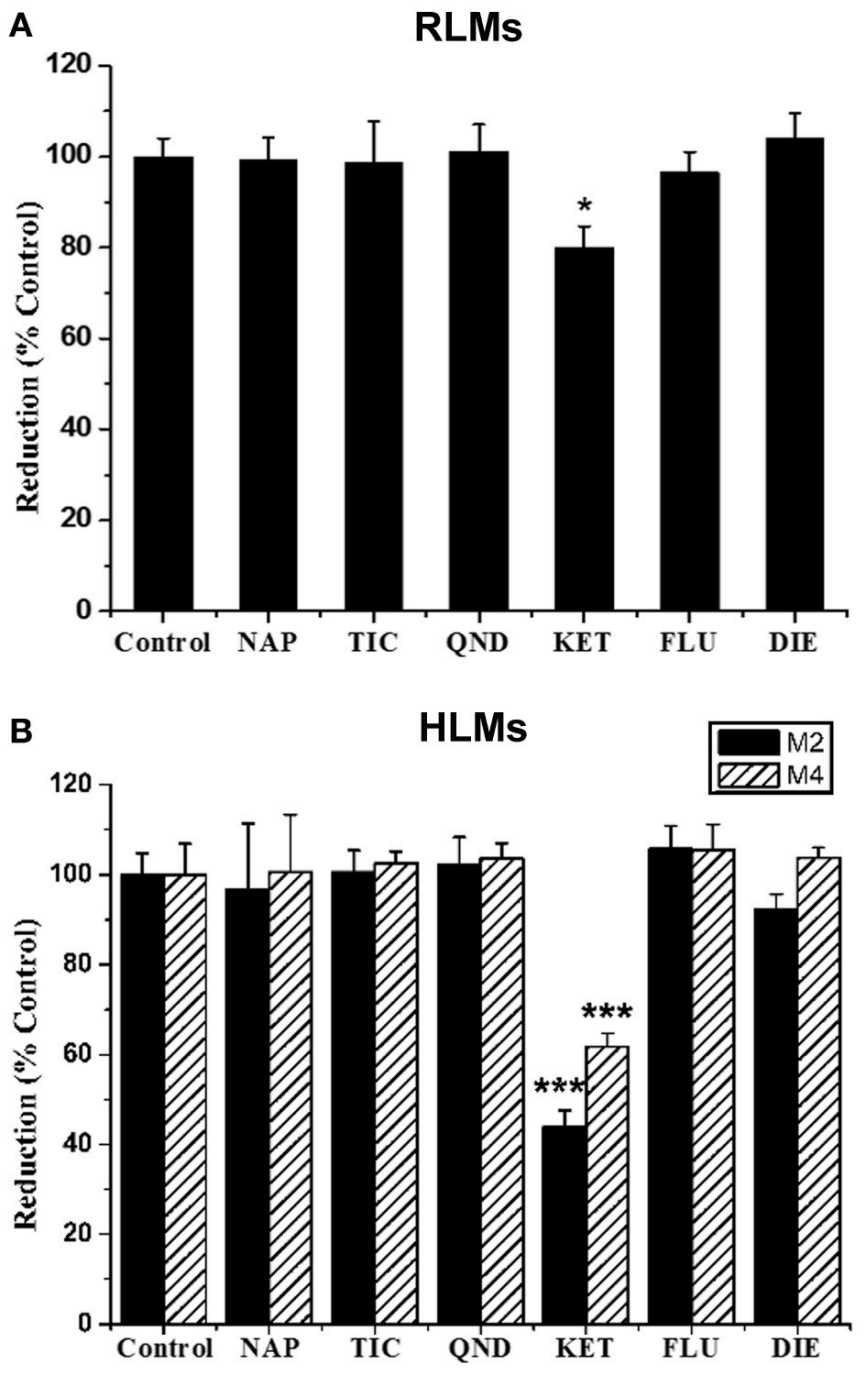
**Effect of CYP inhibitors on the formation of GAA metabolites M2 and M4 in pooled RLMs (A)** or HLMs **(B)**. GAA was incubated with pooled RLMs or HLMs with and without α-naphthoflavone (NAP), ticlopidine (TIC), quinidine (QND), ketoconazole (KET), fluconazole (FLU) and diethyldithiocarbamate (DIE). ^*^p < 0.05, ^***^p < 0.001, compared vs. control. (mean ± SD, *n* = 3).

The effects of six selective inhibitors of CYP enzymes on the formation of M2 and M4 were evaluated *in vitro*, and the results are showed in Figure 7. Ketoconazole (10 μM), a CYP3A inhibitor, could significantly inhibit the formation of M2 in RLMs by 19.91%, and strongly inhibit the formation of M2 and M4 in HLMs by 55.99 and 38.25%, respectively. Other inhibitors could not produce significant effects on the formation of the two metabolites. These data suggested that the formation of M2 and M4 might be mainly catalyzed by CYP3A enzyme.

## Conclusions

GAA could undergo extensive metabolism, including reduction, oxidation, and hydroxylation phase I metabolism, and glucuronidation and sulfation phase II metabolism. Its main metabolic soft spots were 3, 7, 11, 15, 23-carbonyl groups (or hydroxyl groups) and 12, 20, 28 (29)-carbon atoms. The reduction metabolism were catalyzed by CYP3A isoenzyme both in RLMs and HLMs, but with different kinetics. These results will be valuable for understanding the mechanism of pharmacological activities and further pharmacokinetic studies of GAA.

## Author contributions

QC and FC participated in research design. FC, LF, and XT conducted experiments. FC, LY, and LW performed data analysis. QC, FC, and BX contributed to the writing of the manuscript.

## Funding

This study was financially supported by CAMS Innovation Fund for Medical Sciences (CIFMS, 2016-I2M-1-012).

### Conflict of interest statement

The authors declare that the research was conducted in the absence of any commercial or financial relationships that could be construed as a potential conflict of interest.

## Abbreviations

EPI: Enhanced product ion
ER: enhanced resolution
HLMs: human liver microsomes
IDA: independent data acquisition
GAA: ganoderic acid A
GLA: glycyrrhizic acid
MRM: multiple reaction monitoring
RLMs: rat liver microsomes
UDPGA: uridine 5′-diphosphoglucuronic acid
NADPH: β-Nicotinamide adenine dinucleotide phosphate.
