# Supplementary material for: Ganoderic Acid A Metabolites and Their Metabolic Kinetics
Source: Front Pharmacol. 2017 Mar 7;8:101. doi: 10.3389/fphar.2017.00101 (PMC5339268; doi:10.3389/fphar.2017.00101)
Supplement: Supplementary file 1 [file DataSheet1.DOCX]

Supplementary Material

Ganoderic acid A metabolites and their metabolic kinetics

**Fang-Rui** **Cao, Li Feng, Lin-Hu Ye, Li-Sha Wang, Bing-Xin** **Xiao,** **Xue Tao and Qi Chang***

*** Correspondence:** Qi Chang: [qchang@implad.ac.cn](mailto:qchang@implad.ac.cn)

# Supplementary Figures


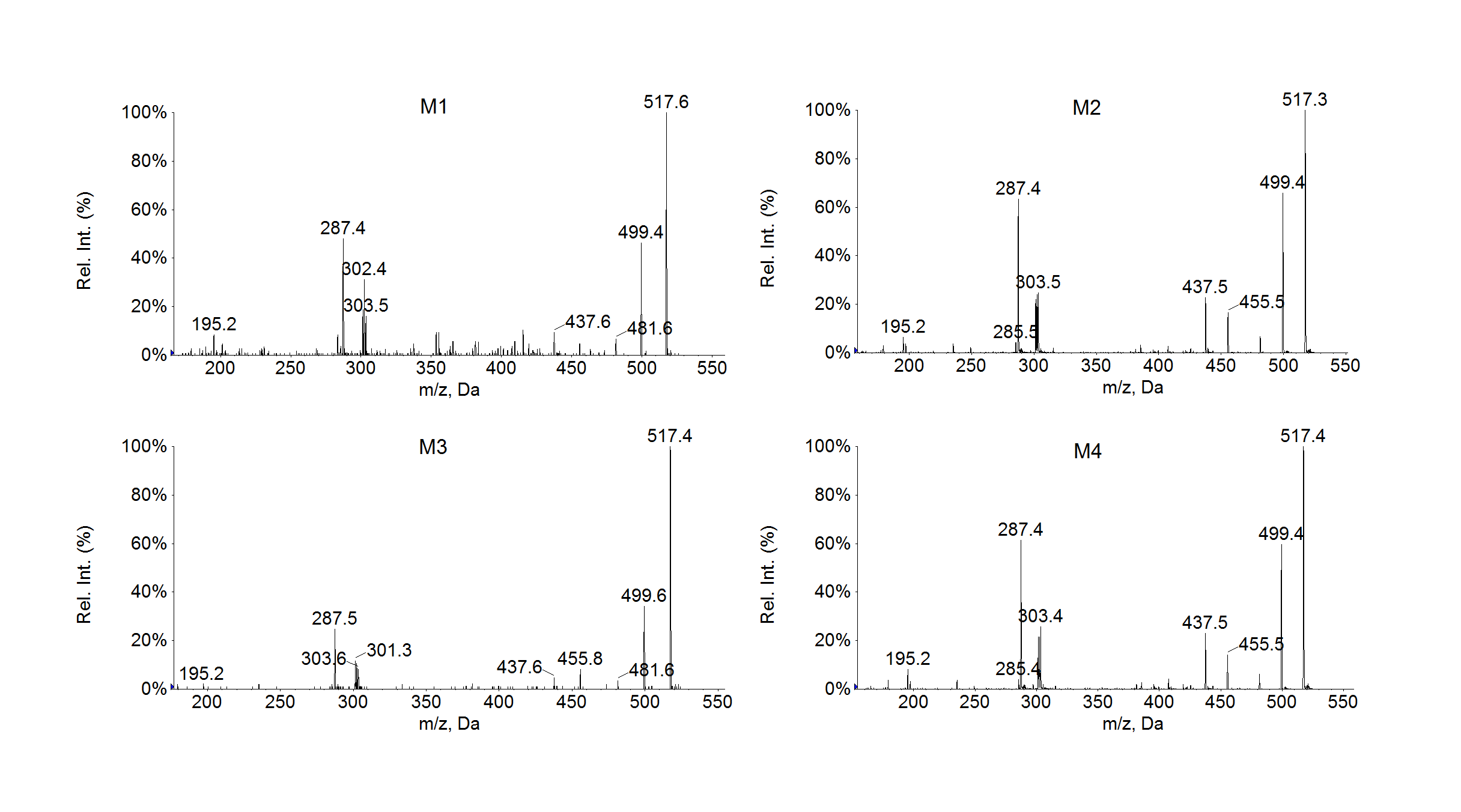


**Supplementary Figure 1.** Mass spectra of metabolites M1-M4.

**
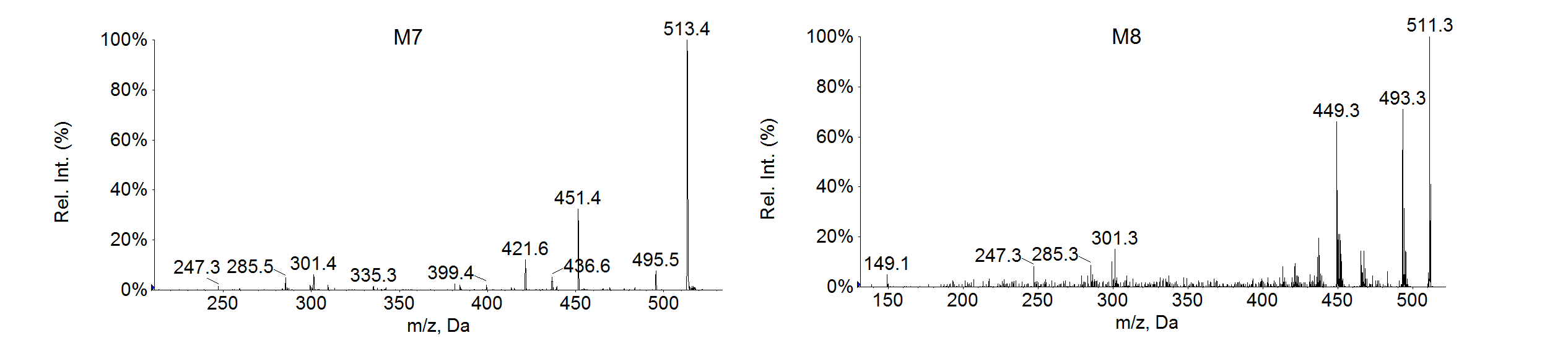
**

**Supplementary Figure 2.** Mass spectra of metabolites M7 and M8.


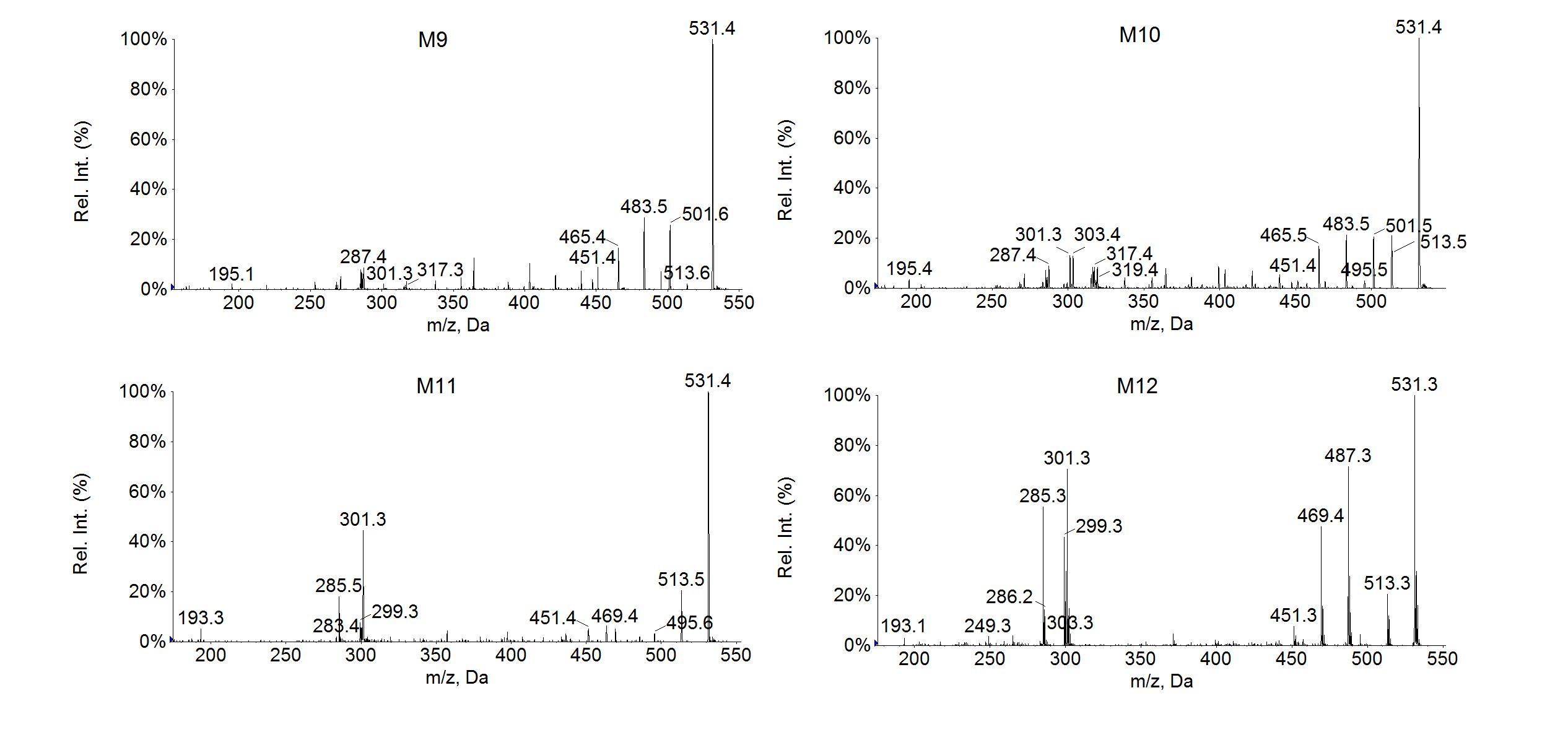


**Supplementary Figure 3.** Mass spectra of metabolites M9-M12.


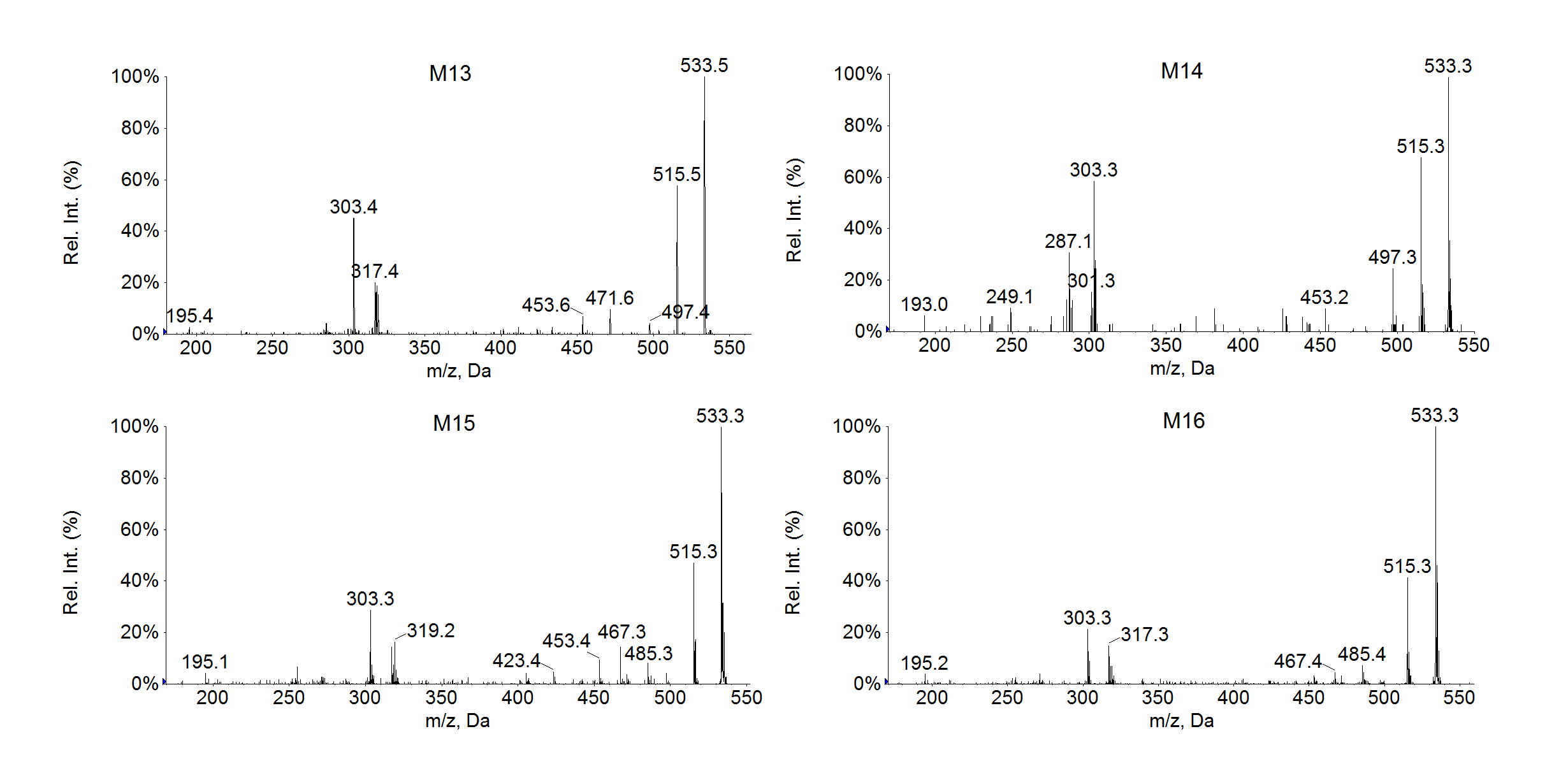


**Supplementary Figure 4.** Mass spectra of metabolites M13-M16.


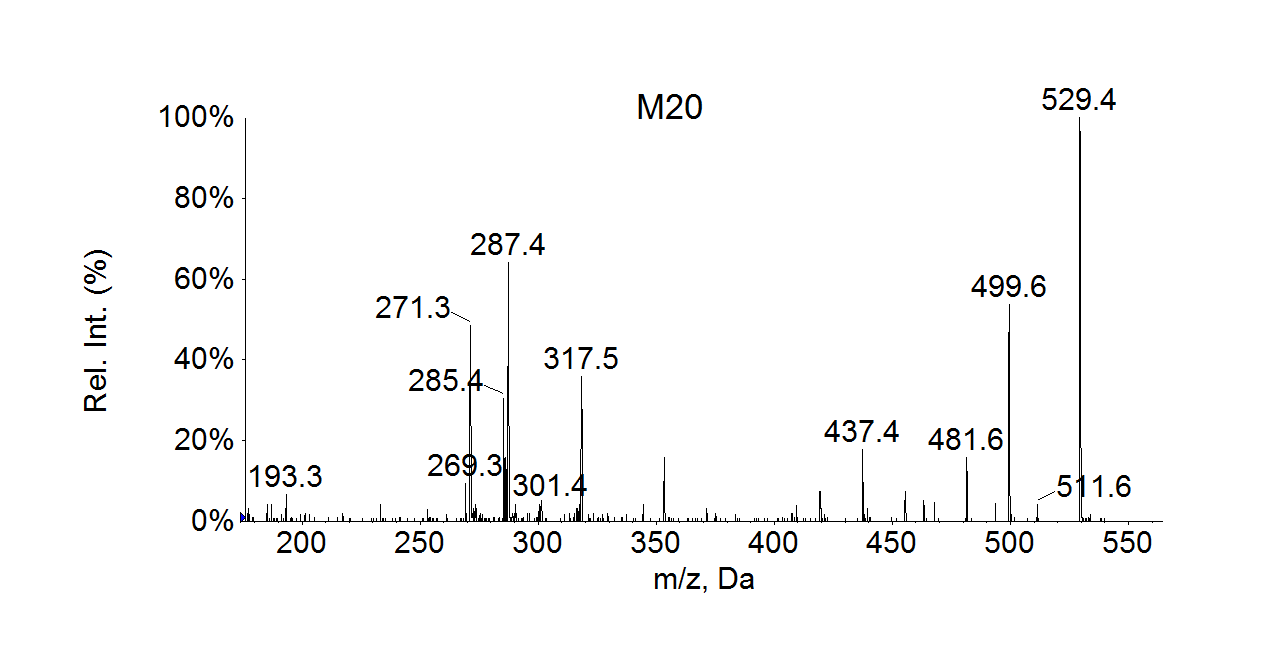


**Supplementary Figure 5.** Mass spectrum of metabolite M20.


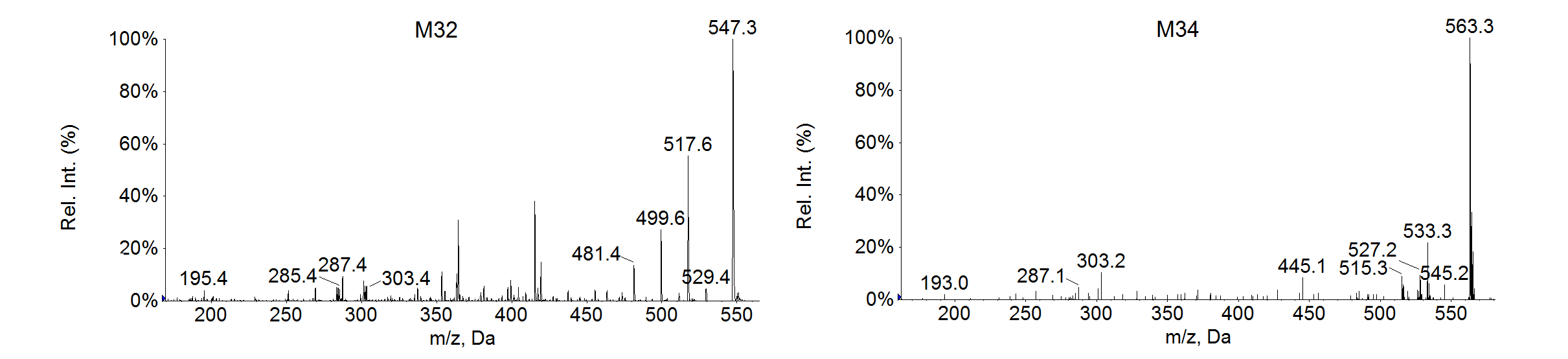


**Supplementary Figure 6.** Mass spectra of metabolites M32 and M34.
